# Synthesis and Characterization of Slow-Release Fertilizer Hydrogel Based on Hydroxy Propyl Methyl Cellulose, Polyvinyl Alcohol, Glycerol and Blended Paper

**DOI:** 10.3390/gels7040262

**Published:** 2021-12-13

**Authors:** Semiu A. Kareem, Idayatu Dere, Daniel T. Gungula, Fartisincha Peingurta Andrew, Abdullahi M. Saddiq, Elizabeth F. Adebayo, Vadlya T. Tame, Haruna M. Kefas, Japari Joseph, David O. Patrick

**Affiliations:** 1 Department of Chemical Engineering, Modibbo Adama University, Yola 652101, Nigeria; Idayatudere@gmail.com (I.D.); hmkefas@mautech.edu.ng (H.M.K.); dopatrick@mautech.edu.ng (D.O.P.); 2 Department of Crop Production and Horticulture, Modibbo Adama University, Yola 652101, Nigeria; dgungula@mautech.edu.ng (D.T.G.); tammeval@gmail.com (V.T.T.); 3 Department of Science Laboratory Technology, Modibbo Adama University, Yola 652101, Nigeria; 4 Department of Soil Science, Modibbo Adama University, Yola 652101, Nigeria; amuhdsaddiq@gmail.com; 5 Department of Agricultural Economics and Extension, Modibbo Adama University, Yola 652101, Nigeria; eadebayo86@gmail.com; 6 Department of Chemistry, Modibbo Adama University, Yola 652101, Nigeria; jjapari75@gmail.com

**Keywords:** hydrogel, blended paper, slow-release fertilizer, hydroxy propyl methyl cellulose, polyvinyl alcohol

## Abstract

In this study, biodegradable slow-release fertilizer (SRF) hydrogels were synthesized from hydroxyl propyl methyl cellulose (HPMC), polyvinyl alcohol (PVA), glycerol and urea (SRF1) and HPMC, PVA, glycerol, urea and blended paper (SRF2). The fertilizer hydrogels were characterized by SEM, XRD and FTIR. The swelling capacity of the hydrogels in both distilled and tap water as well as their water retention capacity in sandy soil were evaluated. The hydrogels had good swelling capacity with maximum swelling ratio of 17.2 g/g and 15.6 g/g for SRF1 and SRF2 in distilled, and 14.4 g/g and 15.2 g/g in tap water, respectively. The water retention capacity of the hydrogels in sandy soil exhibited higher water retention when compared with soil without the (SRFs). The soil with the hydrogels was found to have higher water retention than the soil without the hydrogels. The slow-release profile of the hydrogels was also evaluated. The result suggested that the prepared fertilizer hydrogels has a good controlled release capacity. The blended paper component in SRF2 was observed to aid effective release of urea, with about 87.01% release in soil at 44 days compared to the pure urea which was about 97% release within 4 days. The addition of blended paper as a second layer matrix was found to help improve the release properties of the fertilizer. The swelling kinetic of the hydrogel followed Schott’s second order model. The release kinetics of urea in water was best described by Kormeye Peppas, suggesting urea release to be by diffusion via the pores and channels of the SRF, which can be controlled by changing the swelling of the SRF. However, the release mechanism in soil is best described by first order kinetic model, suggesting that the release rate in soil is depended on concentration and probably on diffusion rate via the pores and channels of the SRF.

## 1. Introduction

The world population is growing rapidly and is expected to reach 9.5 billion by the end of 2050 [1]. Meanwhile industrialization, desertification and land degradation caused by heavy flooding have diminished arable land and consequently threatened global food security [2]. Amidst all these, global food requirements have risen and is likely to double by 2050 [3]. Many steps are being taken to resolve such issues so as to modify and improve our agricultural system, and make it more successful and sustainable. To meet the increasing food demand, the agricultural sector is bound to use even larger quantities of fertilizer that have being demonstrated to have serious undesirable environmental impacts [1]. The use of conventional or mineral fertilizers tends to reach 2.5 million tonnes with subsequent growth of 1.8 percent per year for its demand and supply gap [4].

Despite the use of high quantities of conventional fertilizers, crop utilization is relatively low with subsequent loss to the environment through leaching, denitrification and surface run off [5]. This loss of fertilizer deprives plant of nutrient, increases process cost and causes damage to the environment [6,7,8]. Recently, to overcome the above-mentioned challenges, intelligent fertilizers such as slow or controlled release fertilizers that supply active nutrients in a slow manner are being researched into and to a limited scale, they have been produced and used [6]. Many studies have shown that slow release or controlled released fertilizer (CRF or SRF) can improve the nutrient use efficiency, reduce damage to crop and environment because it is eco-friendly with minimal pollution [9,10,11,12,13].

Initially Sulphur was used for production of SRF [14] but due to its burst effect, inconsistent result, high cost due to additional requirement of sealants, plasticizers and binders it has been discontinued [5,15]. However, in place of synthetic polymers, eco-friendly biopolymer such as starch, lignin and cellulose were used as alternative materials for implementation of SRFs [16,17,18]. Cellulose is mainly found in high amount in different plant sources where it is cheap, abundantly available and biodegradable [19]. A lot of effort has been made to replace synthetic polymers with cellulose derivatives, which are used in controlled release for drugs [20,21,22], removal of pharmaceutical pollutants in waste water [23,24], dye removal in waste water [25,26] and agrochemicals after crosslinking [27]. Hydroxy propyl methyl cellulose (HPMC) is an odourless, tasteless, white to slightly off white fibrous or granular free flowing powder. It is prepared by modification of alkali cellulose from purified wood pulp treated with 18% sodium hydroxide solution reacted with methyl chloride and propylene oxide to obtain the methyl and hydroxyl ether group, and is widely used in food, drugs and dietary supplement industries [28]. It is an ideal polymer for film coating, which might be used as a basis for hydrophilic matrices for controlled release of drug and fertilizer delivery [29]. 

Polyvinyl alcohol (PVA) has also been studied for used in fertilizer and drug release, since at high content it can control the process release. PVA is a water soluble, semi-crystalline, nontoxic polymer with good physical properties, high ability to absorbed fluid and degrades slowly [30]. It has numerous applications in agricultural industry to deliver fertilizers, pesticide, herbicide [31], extensively used in paper coating, textile sizing, drug release and flexible water-soluble packaging films [30]. Enayatifard et al., [32] studied the effects of HPMC and ethyl cellulose content on release profile and kinetics of diltiazem HCl from matrices. The results showed that these polymers slowed down the release of diltiazem HCl from the matrices and could potentially be used for controlled delivery of highly soluble drugs.

HPMC was also used as binder in the preparation of encapsulated urea kaolinite-controlled release fertilizer [9]. Mela and Darajo, prepared controlled release fertilizers using HPMC layered tablets modified with chitosan and xanthan as matrices [33]. Caccavo et al., produced and characterized hydrogel based granular photo strengthener for prolonged release of fertilizer [34]. PVA and glycerol have been studied for used in controlled fertilizer release [30,35]. Paper is a mat of cellulose fibers that have been beaten in the presence of water collected on a screen and dried [36]. A paper base typically contains 90–99% cellulose fibers, which are the primary structural elements influencing end use properties. Paper network is composed of randomly laid fibrous cellulose and non-fibrous (filler) materials. Its complicated set of cavity pore channels having capillary dimensions make it readily permeable to liquids [37]. Hence its structure can be modified during contact with liquids because it disrupts hydrogen bonds, relaxes fibers and produces dimensional changes in pores and capillaries [37]. The use of plain waste paper blended with HPMC, PVA and glycerol to formulate a second layer coating for controlled release of fertilizer is an important concept which has not been reported in literature. Furthermore, the use of paper implies the possibility of recycling waste paper thus reducing pollution due to paper disposal. In addition, incorporation of paper in the coating makes it biodegradable. The novelty of using paper is its biodegradability so that after serving its purpose, it can be degraded without constituting another environmental nuisance.

In our continuous effort [38] to design materials that can effectively deliver urea to plants in a controlled manner, we present here the synthesis of SRF hydrogel based HPMC/PVA/Glycerol with blended paper as the second layer. Furthermore, the effects of the blended paper on release, swelling and water retention in soil were investigated.

## 2. Results and Discussions

### 2.1. Characterization

#### 2.1.1. Surface Morphology Analysis

**Figure 1:**
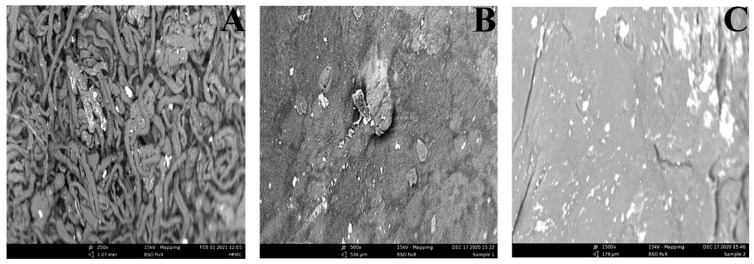
SEM Images of HPMC (**A**) SRF1 and (**B**) SRF2 (**C**).

SEM technique was used to observe the surface morphologies of the hydrogel (SRF1, SRF2) and the starting materials (HPMC), and the results are presented in Figure 1. From Figure 1A, it can be seen that HPMC powder is a porous spongy particle like materials which has a rough, less dense surface that is no more recognizable in both hydrogels (SRF1 and SRF2) because of adsorption of urea fertilizer to its surface (microstructure). This porous nature aids to increase the surface area and can enhance the adsorption capacity of HPMC by yielding more sites for more interaction. Figure 1B (SRF1) has a uniform and smooth surface without any crack except for the existence of a defect on the surface. The SRF2 (Figure 1C) has a rough packed surface having no porous cracks on the surface, which may have enhanced its water uptake and retention and consequently the release capacity. The dense and compact structure of the hydrogel is due to the co-polymerization of the polymer and the composite blends resulting from the crosslinking and incorporation of the paper blends into the matrix. This is because polymer matrices can either be chemically cross-linked through covalent or hydrogen bonds [39]. 

#### 2.1.2. FTIR Spectra 

**Figure 2:**
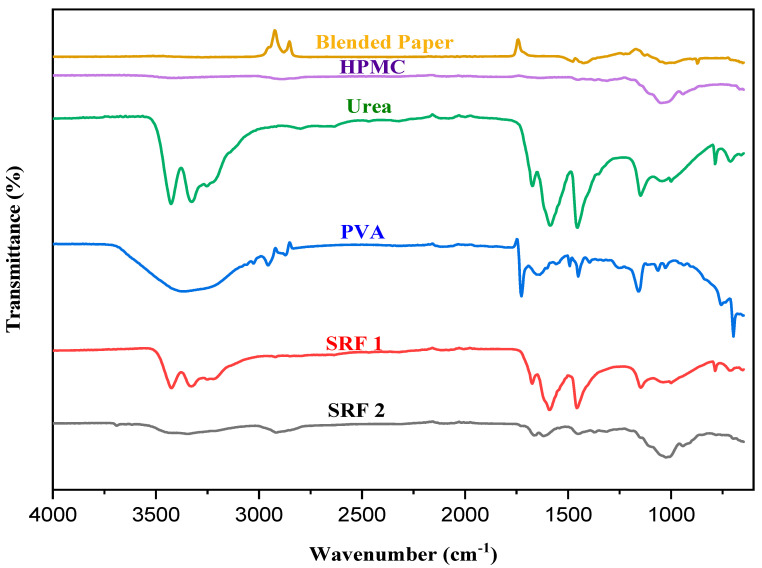
Overlay FTIR spectra of Blended paper, HPMC, Urea, PVA, SRF 1 and SRF 2.

The FTIR spectra of the blended paper (BP), HPMC, PVA, Urea and SRF’s (SRF1 and SRF2) are shown in Figure 2. In the spectrum of blended paper, peaks at 3272 cm^−1^ and 1028 cm^−1^ are associated with stretching vibration of O-H and C-O-C in C-C out of plane stretching vibration [37]. The peaks at 2759 cm^−1^ and 2114 cm^−1^ are assigned to –CH– in CH_2_ stretching vibration. The absorption band at 1425 cm^−1^ is ascribed to –CH_2_– stretching. In the spectrum of HPMC absorption bands at 3424 cm^−1^ is ascribed to the stretching vibration of O-H. The peak at 2887 cm^−1^ represents the stretching of CH_2_, and C=O stretching is observed at 1648 cm^−1^. The band at 1452 cm^−1^ represents CH_2_ and 1048 cm^−1^ is assigned to C–O–C stretching [40]. In the spectrum of PVA, characteristic peaks of PVA occurred at 3358 cm^−1^ is assigned to –OH stretching vibration of hydroxyl group in alcohol, 2956 cm^−1^–2873 cm^−1^ characterized the aliphatic –CH symmetric stretching vibration. 1726 cm^−1^, 1450 cm^−1^ and 1158 cm^−1^ correspond to C=O carbonyl, -CH_2_, and C–O, C–O–C [30]. In the spectrum of Urea, two peaks at 3426 cm^−1^ and 3326 cm^−1^ are related to the stretching vibration of –NH and –OH group in urea. Furthermore, peaks appeared at 2799 cm^−1^, 1586 cm^−1^, 1453 cm^−1^, 1148 cm^−1^ and 1001 cm^−1^, indicating the –CH_2_ stretching vibration, –NH angular deformation, CH stretching in –CH_2_ aliphatic group and –C–O stretching in –C–O–C group. In the spectrum of SRF1 and SRF2 new peaks appeared around 3425 cm^−1^ and 3328 cm^−1^ in SRF1, and around 3689 cm^−1^, 3616 cm^−1^, and 3345 cm^−1^ in SRF2 after incorporation of urea into the hydrogel. These peaks may be attributed to the formation of intermolecular hydrogen bond between –OH group of HPMC and the carbonyl group of PVA and urea, which confirms the encapsulation of the urea in the matrix via physical entrapment and its interaction with the polymer (30). This interaction was indicated by the shifting of peaks to higher wavelengths and the carbonyl group of (PVA and HPMC) [41], and as well due to the presence of –NH_2_ that is part of O=C–NH_2_ functional group in urea [42]. Most of the characteristic peaks shifted to higher frequencies in both hydrogels (SRF1 and SRF2) because the hydrogen bond between water and glycerol was replaced with hydrogen bond between HPMC and glycerol which is weak [30]. The appearance of urea characteristic peaks in FTIR spectra of SRF1 and SRF2 confirms the successful encapsulation of urea fertilizer compound within the matrix hydrogel.

#### 2.1.3. XRD Analysis

**Figure 3:**
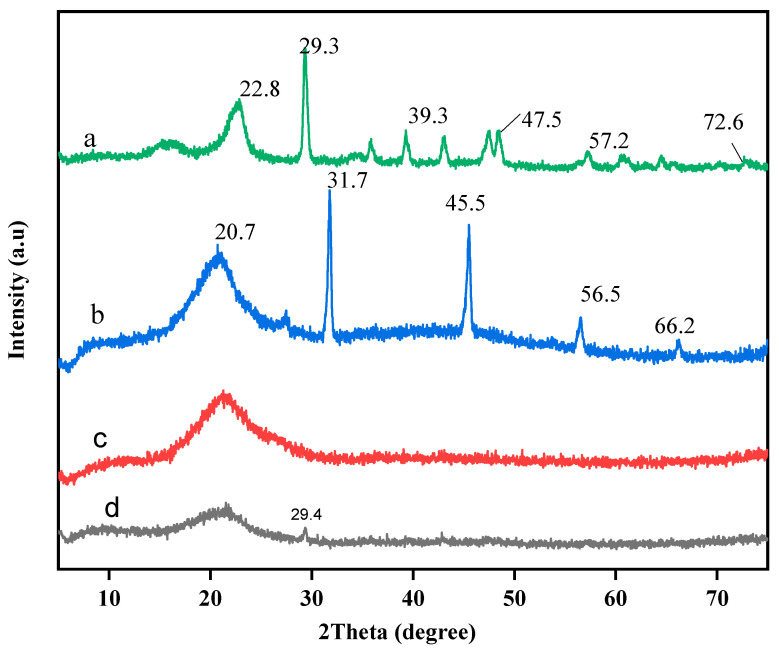
XRD patterns of blended paper (**a**), HPMC (**b**), SRF1 (**c**), SRF2 (**d**).

Figure 3 shows the XRD patterns of blended paper, HPMC and SRF. The characteristic diffraction peaks of the blended paper are shown at 2θ = 22.8°, 29.3°,39.3°, 47.5°,57.2° and 72.8°. The XRD pattern of HPMC presents diffraction peaks of cellulose at 20.7°, 31.7°, 45.5°,56.5°, 66.2° indicating that HPMC is crystalline with crystalline peak. As it can be seen, all the samples exhibit typical cellulose diffraction peaks [43]. In the pattern of SRF2, a shift in the basalt diffraction peak from 29.3 to 29.4 was observed but became weaker. This might be due to high compatibility between the blended paper, HPMC that led to the insertion of paper into HPMC layers which occurred as a result of cationic exchange between hydrogen bond and COO [44]. This slight change indicates that urea was absorbed into the HPMC-blended paper matrix, which slowed the release of the nutrient. However, most of the characteristic peaks of blended paper and HPMC were seen to disappear, while in SRF1 no peak was observed with disappearance of characteristic peaks of HPMC. Similarly, a broad peak of amorphous structure of polymer present shows the compositions of organic materials formed and that the blended paper was dispersed in SRF [45].

### 2.2. Swelling Studies of the Hydrogels in Different Solutions 

**Figure 4:**
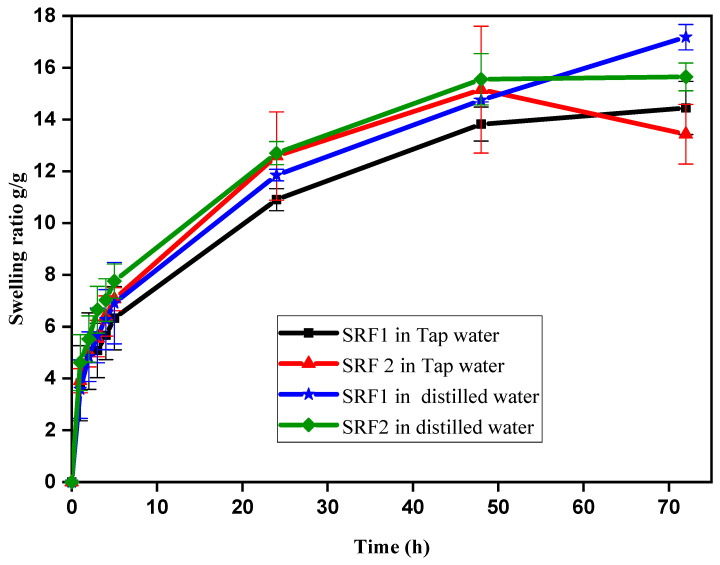
Swelling behaviors of hydrogels in solution.

Water absorption is one of the most important characteristics of SRF hydrogels [45]. Figure 4 shows the water absorption in distilled and tap water. The results showed that the swelling ability in distilled water was higher compared to tap water, this could be as a result of the increase in the osmotic pressure difference between the polymeric network and immersion medium. According to Donnan equilibrium theory, osmotic pressure is the driving force for swelling in hydrogel. The result of this study is consistent with the findings of Enayatifard et al., which showed that the presence of a highly water-soluble compound in HPMC matrix generated an additional osmotic gradient, thereby resulting in a faster rate of polymer swelling and a large increase in gel thickness [32]. 

Similarly swelling degree might as well be influenced by the ions present in the swelling medium, where by the presence of these ions (Mg^2+^, Ca^2+^ and Na^+^) increased the ionic crosslinking density of the matrix blend causing a decrease in water absorption as reported by [45]. This observation is common in ionic hydrogels static swelling experiments [46]. The swelling equilibrium of the SRF’s hydrogel in both medium was attained at 72 h except SRF2 in tap water which attained its equilibrium at 48 h with swelling rate of 17.2 (g/g) and 15.6 (g/g) for SRF1 and SRF2 in distilled water while 14.4 (g/g) and 15.2 (g/g) for SRF1 and SRF2 in tap water respectively. The swelling rate of SRF2 compared to SRF1 in distilled and tap water was high. This might be as a result of the additional hydroxyl group found in network of cellulose paper, since paper network contains randomly laid cellulose fibrous and non-fibrous (filler) materials which contains complicated set of cavity pore channels with variety of capillaries dimension, which make its readily permeable to liquids [37].

### 2.3. Swelling Kinetics

**Figure 5:**
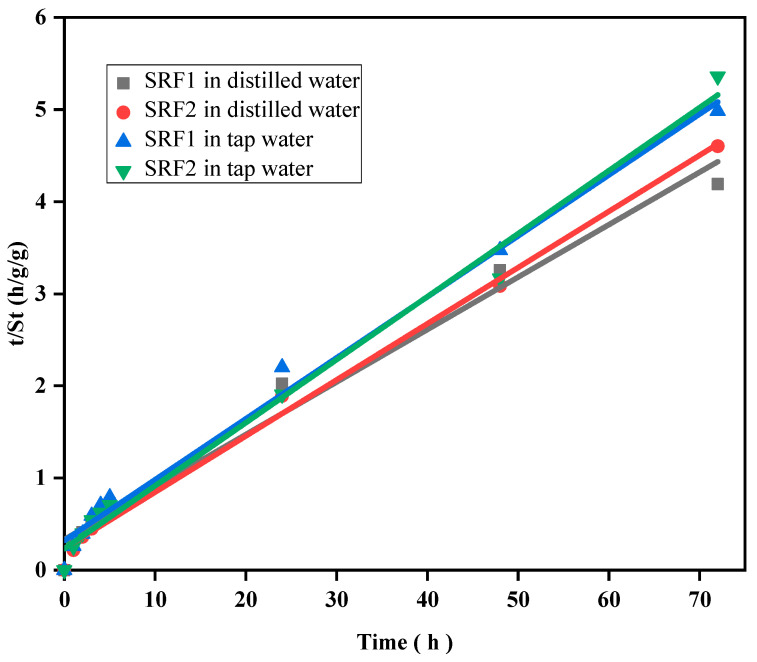
Swelling kinetics of hydrogel in solution.

The swelling kinetics of the hydrogels in tap and distilled water was determined and the result is shown in Figure 5. A straight-line graph was obtained from the plot of *t*/*St* versus *t* with a good linear correlation coefficient as given in Table 1. *S_eq_* and *K_is_* were calculated from the slope and intercept obtained from fitted straight lines. The result shows that the swelling behavior of hydrogel in distilled and tap water matched the Schott’s second order swelling kinetic model. The data presented for *S_eq_* and *K_is_* in tap water were lower compared to that in distilled water.

### 2.4. Water Retention Properties of the SRF Hydrogel in Soil

**Figure 6:**
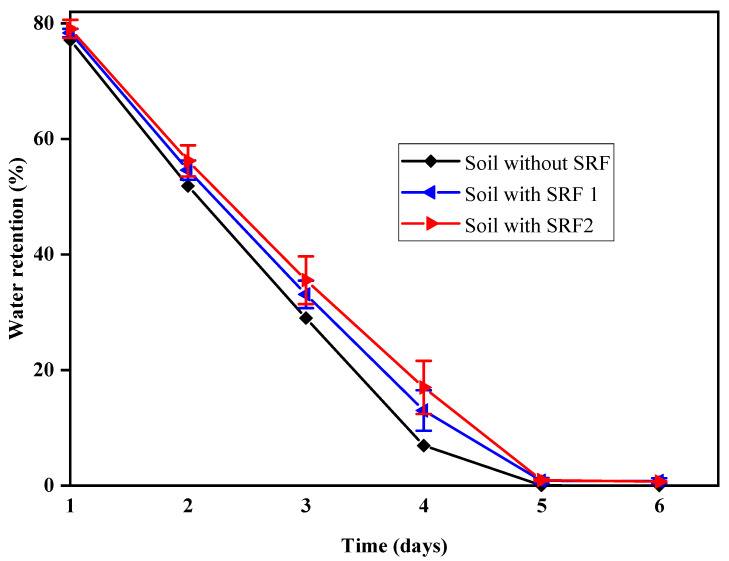
Water retention behaviour of sandy soil with and without hydrogel.

Water retention of hydrogel is an important feature of SRF in soil. Figure 6 shows the water retention behaviour of sandy soil with and without SRF. By adding the hydrogel in soil, the water retention increased for some time while the water content of soil without the SRF completely vaporized after the 6th day. The water retention of soil containing SRF2 was high compared to the soil with SRF1. The water retention ratio of soil without SRF hydrogel remained 51.9 and 0.04 on the 2nd and 5th days, respectively, while that with SRF1 and SRF2 remained 54.6, 0.8 and 56.2, 1.0, respectively. A similar phenomenon was also observed by others [47]. This study finds out that the addition of blended paper as second layer in SRF2 helps in increasing the water retention in soil and decreases water transpiration. That is, it withholds sufficient water, which would aid its efficient utilization for crop cultivation thereby minimize irrigation water for farming.

### 2.5. Release Profile of SRF Hydrogels and Their Kinetics of Release

**Figure 7:**
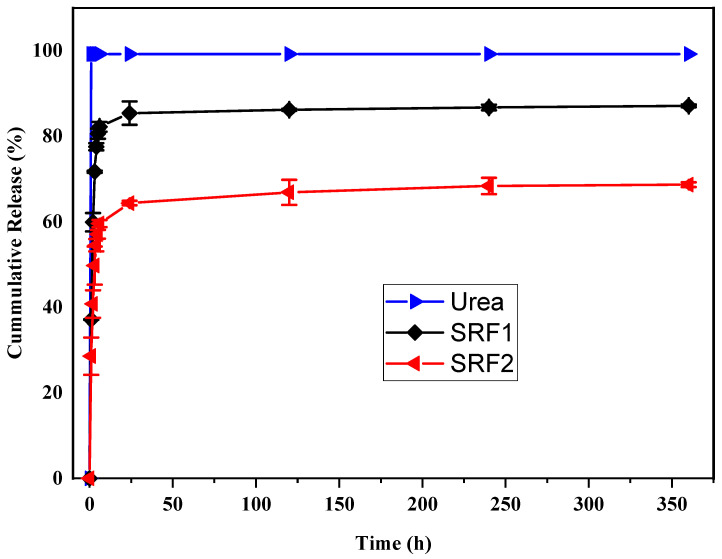
Release behavior of urea, srf1 without paper and srf2 with blended paper in water.

**Figure 8:**
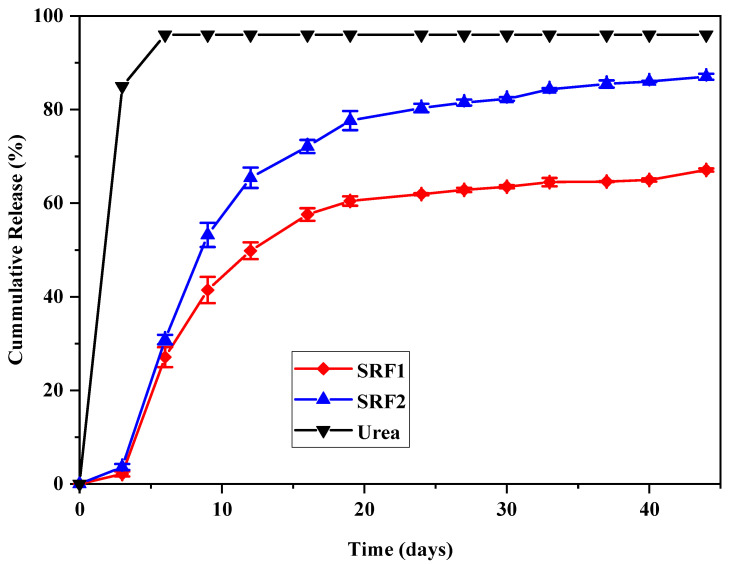
Kinetic parameters of each of the model used.

The cumulative release rate of pure urea and the hydrogel fertilizers; SRF 1 and SRF2 in water and soil is shown in Figure 7 and Figure 8, respectively. The result indicates 99% and 97% release of pure urea used as control in water and soil within one hour and four days, respectively. The urea release in distilled water; being a static medium was faster compared to that in soil. A cumulative release of approximately 37%, 82.2% and 85.4% were observed for SRF1 in water at 1, 6 and 24 h, while 28.6%, 59.6% and 64.4% were observed for SRF2 at the same time in water. The result suggests that the blended paper and HPMC used as second layer coating may be responsible for slower release observed in SRF2 compared to SRF1. The blended paper in the matrix might have created a physical barrier in the form of membrane resistance of the matrix, thereby withstanding osmotic pressure, and causing a slower release by diffusion as a result of the concentration or pressure gradient or both [37,48,49]. The cumulative urea release in soil is shown in Figure 8, the release is significantly lower than that in water. The sustained release rate observed in soil is expected, unlike in distilled water which is static the soil medium is dynamic. For SRF1 cumulative release rates of 27.1%, 64.5% and 67.1% were observed at 6, 30 and 40 days respectively. The cumulative release of urea in SRF1 in soil reaches 67.1% at 44 days. Comparatively, SRF2; the hydrogel with blended paper and HPMC as second layer coating had a higher cumulative percentage release of 30.8%, 82.3% and 87.0% at 6, 30 and 44 days, respectively. The blended paper in SRF2 might have aid absorption of water thereby dissolving the urea in the matrix and consequently resulted in maximum release of 87% of the encapsulated urea at 44 days compared to 67.1% of urea in SRF1 at 44 days. 

Four different kinetic models namely; Korsmeyer–Peppas, Higuchi, Zero and First order kinetic models were applied to analyze the cumulative release data in both water and soil. The kinetic release parameters in water and soil are presented in Table 2 and Table 3, respectively and the plots for all the kinetic models is attached in supplementary document (Figures S1–S16). From the tables it can be seen that Korsmeye peppas kinetic equation is the most suitable model to describe the release mechanism of fertilizer in water (R^2^ > 0.85) K × 10^2^ is 0.5 and n > 0.45. This suggest that urea release was by diffusion via the pores and channels of the SRF, which can be controlled by changing the swelling of the SRF [45]. However, the release mechanism in soil is best describe by first order kinetic model (R^2^ > 0.73), suggesting that the release rate of urea in soil is dependent on its concentration [45]. Reports from previous studies [50,51,52] revealed that release from any loaded material from polymeric excipient could be physically due to diffusion, polymer degradation and dissolution or polymer swelling. 

## 3. Conclusions

In this work, slow release and water retention fertilizer hydrogels from hydroxyl propyl methyl cellulose (HPMC)/polyvinyl alcohol (PVA)/glycerol were prepared with blended paper as a second layer matrix which encapsulated urea fertilizer. The hydrogels were analyzed by SEM and XRD. The water absorption in different media and water retention in soil were calculated. The SEM and XRD characterizations confirmed that urea had been successfully absorbed or encapsulated within layer of the matrix, and paper had been introduced into the fertilizer. The swelling behavior of SRF was studied in distilled and tap water. The SRF2 hydrogel had water absorbency of 15.6 (g/g) and 15.2 (g/g) in distilled and tap water, respectively. The water retention was found to increase in soil with SRF hydrogels. Similarly, the slow-release behavior of urea was studied in distilled water and soil. As expected, the swelling ability in distilled water was higher compared to tap water. The swelling kinetics of the hydrogel followed Schott’s second order equation. The cumulative release fraction of urea was slower in soil compared to that in water. The release kinetic of urea follows Korsmeye peppas kinetic equation in water (R^2^ > 0.85) and first order kinetic model (R^2^ >0.56) in soil. These results suggest that the hydrogel SRF from HPMC/PVA/Gly with paper as second layer will go a long way to improve agricultural and horticultural productivity through efficient fertilizer utilization and improved water conservation thereby reducing the amount of urea fertilizer and water for irrigation purposes.

## 4. Materials and Methods

### 4.1. Materials

Hydroxy propyl methyl cellulose (viscosity 25–100 mpa.s) was purchased from Shanghai Chemical Factory, Shanghai, China. Polyvinyl alcohol (degree of hydrolysis, 87–89%, Viscosity of 4% aqueous solution at 20 °C, 35–45 cP) and glycerol were purchased from Guangdang Guanghua Chemical Factory, co ltd, Shanfai, China and Kermel. A4 Paper was obtained from a business center Modibbo Adama University commercial center Yola, Nigeria. Thiosemicarbazide was purchased from Kermel, Tianji, Jinnan District China, deacetyl monoxime was purchased from Blue Horizone, Gopal Niwas, Mumbai, Maharashtra, India, phosphoric acid and sulfuric acid were obtained from commercial outlet; Sigma Aldrich, St. Louis, MO, USA and British Drug Houses, London, UK. All chemicals’ reagents were of analytical grade and were used without further purification. Distilled water was obtained from Chemical Engineering laboratory, Modibbo Adama University, Yola, Nigeria and was used throughout the experiment.

### 4.2. Preparation of SRF Hydrogel Based on HPMC, PVA, Glycerol with Paper as Second Layer

Calculated amount of HPMC, PVA and glycerol were mixed at various ratio and 50 mL distilled water was slowly added to the mixture at room temperature under magnetic stirring for 20 min to form dispersion. When completely mixed, the temperature of the mixture was slowly raised to 90 °C maintaining stirring for another 5 min to completely gelatinize the HPMC. Two grams (2 g) of urea fertilizer was thoroughly blended with the mixture with glass rod, there after it was dried at 25 °C forming a single layer matrix. The above procedure was repeated and blended with 0.5 g of paper as the second layer and as well without paper as the second layer. Paper was milled into finer particles by soaking the plain paper in distilled water and was blended using a blender until a homogenous mixture was obtained. The mixture was sieve and air dried. The dried paper was later ground into smaller particles and was used in the matrix formulation. Calculated amount of HPMC, PVA and Glycerol were mixed at ratios given in Table 4, 50 mL distilled water was slowly added to the mixture at room temperature under magnetic stirring for 20 min to form dispersion. The temperature was slowly raised to 90 °C maintaining stirring for 5 min to completely gelatinized the HPMC. Then 0.5 g of the blended paper was added and thoroughly mixed and applied on the first layer forming the second layer and was dried at room temperature before it was later oven dried at 50 °C for 4 h. The matrix was later dried at room temperature and then oven dried at 50 °C for 4 h. Table 4 contains the matrix formulation as reported by Sofyane et al., [30] but with a small modification.

### 4.3. Characterization

The surface morphology of the prepared hydrogels was observed by scanning electron microscope PRO:X: Phenonm World (Model:800-07334), Eindhoven, Netherlands with backscattered electrons detector (BSD), electrons beam voltage of 15 kV. The FTIR spectra of the HPMC, PVA, blended paper and hydrogels matrix were measured by Agilent cary 600 series, Santa Clara, CA, USA at wavelength of 400 to 4000 cm^−1^. X-ray diffraction patterns of HPMC, paper and SRF hydrogel were recorded by X-ray diffractometer, thermoscientific (Model: ARL X’TRA), Eindhoven, Waltham, USA in the 2*θ* range of 10° to 70°. Urea content in solution was determined by ultraviolet spectrophotometry (Jenway 6705), Shanghai, China based on standard curve of urea obtained using reaction of urea with diacetylmonoxim colorimetric method [9].

### 4.4. Swelling Behavior of Hydrogels

About 0.5 g of dried samples were weighed, enclosed in 200 mesh bags and immersed in distilled and tap water at 37 °C. After regular time intervals, the swollen samples were removed from the water, dried off of any excess water with a filter paper and weighed. The procedure was replicated three times for SRF1 and SRF2. The water uptake was calculated according to Equation (1).

(1) $$W\cdot A=\frac{M-M_{j}}{M_{j}}$$

where, *M* and *M_j_* are weights of swollen and dried samples, respectively.

#### Swelling Kinetics

The Swelling kinetics of the matrix was determined using Schott’s second order swelling kinetic model given as

(2) $$\frac{t}{S_{t}}=\frac{1}{K_{is}}+\frac{1}{S_{eq}}t$$

where, *S_t_* (g/g) is the water absorption capacity at any time *t* (h), while *S_eq_* (g/g) stands for the theoretical equilibrium water absorption and *K_is_* (g/g) represents a constant for the initial swelling rate. From the plot of *t*/*St* versus *t, a* straight line was obtained, which shows that the swelling obeys Schott’s second order model and the values of *S_eq_* and *K_is_* were obtained from the slope and intercept.

### 4.5. Water Retention of Hydrogels in Soil

Dried samples (0.5 g) were weighed and added into 100 g of dried sandy soil in a 250 mL beaker. At the same time, 100 g of dried sandy soil without the hydrogel was put in another beaker as control. Then 50 mL of distilled water was added to each beaker was and weighed. The beakers were weighed every day at ambient temperature (at 37 °C) until it got to a constant mass. The water retention Wr (%) of soil was calculated by Equation (3). Three different sample for SRF1 and SRF2 were used.

(3) $$W_{r}=\frac{W_{i}-W}{W_{o}-W}\times 100$$

where, *W_r_* is the water retention rate and *W* is the total mass of sandy soil and beaker. *W_o_* is the total mass of sand, beaker and sample after adding distilled water. *W_i_* is the total mass of sand, beaker, and sample at regular intervals.

### 4.6. Slow-Release Behavior of Hydrogels

The release behavior of the matrix was first studied in distilled water, where 1 g of dried sample was immersed in a 250 mL beaker placed in a tight container of 200 mL distilled water maintained at 37 °C. At each preset time interval, the samples were transferred into another beaker containing 200 mL fresh water, this continued until all the urea was released. Then 1 mL of the solution was withdrawn from the beaker and the urea content in the solution was determined by UV-Vis Spectrophotometer using the diacetylmonoxime calorimetry method based on the standard curve obtained. Three samples of SRF1 and SRF2 were used.

### 4.7. Release Kinetic of Hydrogel

The release behavior was analyzed by four different empirical equations that is the power equation by Korsmeyer-Peppas, Higuchi, zero order and first order mathematical models. The power equation is given as Equation (4).

(4) $$\frac{M_{t}}{M_{\infty }}=K_{i}t^{n}$$

where *K_i_*, *t*, and *n* are diffusion content, time and diffusion exponent indicative of the release mechanism, respectively. *M_t_/M_∞_* is the fraction of fertilizer released at time *t*. Nutrient release mechanism is classified according to different values of *n*, if *n* < 0.45, then it is Fickian diffusion mechanism; if 0.45 < *n* < 0.89 then is non Fickian diffusion release; if *n* = 1 then is zero order release, if *n* > 0.89 then is case II transport [42]. The Higuchi kinetic model can be written as Equation (5).

*Q_t_* = *K_H_t*^1/2^ (5)

The Zero order kinetic model is given as follows

*Q_t_* = *K_o_t* (6)

The First order model is given as below

*Q_t_* = *Q_o_e*^−kt^ (7)

where *Q_t_* is the amount of nutrient release at time *t*, *Q_o_*, is the amount of nutrient initially in the matrix, *t* is the time, *K_H_* is the Higuchi dissolution constant, *K_o_* and *K* are the zero order and first order release constant, respectively.

## Figures and Tables

**Table 1:** Swelling kinetic parameters for the hydrogels in distilled and tap water.

Conditions	Parameters	SRF1	SRF2
Distilled water Tap water	S_eq_ (g/g)	17.56	16.40
	K_is_ (g/g)	3.00	4.23
	R^2^	0.98052	0.98955
	S_eq_ (g/g)	15.11	14.61
	K_is_ (g/g)	3.12	4.32
	R^2^	0.98909	0.99373

**Table 2:** Kinetic parameters from different model for fertilizer release in water.

Kinetic Models	Parameter	SRF1	SRF2
Korsmeyer–Peppas	R^2^	0.85101	0.71925
	n	0.6842	0.5027
	K × 10^2^	0.50	0.45
Higuchi model	R^2^	0.85108	0.71925
	K × 10^2^	0.19	0.14
Zero order model	R^2^	0.85108	0.71925
	K × 10^2^	0.19	0.14
First order model	R^2^	0.5958	0.6686
	K × 10^−2^	0.95	0.81

**Table 3:** Kinetic parameters from different model for fertilizer release in soil.

Kinetic models	Parameter	SRF1	SRF2
Korsmeyer–Peppas	R^2^	0.4317	0.5329
	n	1.2404	1.1599
Higuchi model	R^2^	0.4317	0.5359
	K × 10^2^	0.745	0.865
Zero order model	R^2^	0.4317	0.5359
	K × 10^2^	0.745	0.865
First order model	R^2^	0.5670	0.7314
	K × 10^2^	0.745	0.865

**Table 4:** Composition of the Hydrogels.

Sample	HPMC (g)	PVA (g)	Gly (g)	Paper (g)
SRF1	5.0	0.4	0.5	-
SRF2	5.0	0.4	0.5	0.5

## Supplementary Materials

The following are available online at https://www.mdpi.com/article/10.3390/gels7040262/s1, Figure S1: Graph of Zero order kinetic model of ln Qt vs. ln t for SRF1 in water, Figure S2: Graph of Zero order kinetic model of ln Qt vs. ln t SRF2 in water, Figure S3: Graph of Higuchi model of ln Qt vs. ln t0.5 for SRF1 in water, Figure S4: Graph of Higuchi model of ln Qt vs. ln t0.5 for SRF2 in water, Figure S5: Graph of Korsmeyer-Peppas of ln Mt/ M∞ vs. ln t for SRF1 in water, Figure S6: Graph of Korsmeyer-Peppas of ln Mt/ M∞ vs. ln t for SRF2 in water, Figure S7: Graph of First order kinetic model of ln Qt vs. Time for SRF 1 in water, Figure S8: Graph of First order kinetic model of ln Qt vs. Time for SRF 2 in water, Figure S9: Graph of Zero order kinetic model of ln Qt vs ln t for SRF1 in soil, Figure S10: Graph of Zero order kinetic model of ln Qt vs. ln t for SRF2 in soil, Figure S11: Graph of Higuchi model of ln Qt vs ln t0.5 for SRF1 in soil, Figure S12: Graph of Higuchi model of ln Qt vs. ln t0.5 for SRF2 in soil, Figure S13: Graph of First order kinetic model of ln Qt vs Time for SRF 1 in water, Figure S14: Graph of First order kinetic model of ln Qt vs. Time for SRF 2 in water, Figure S15: Graph of Korsmeyer-Peppas of ln Mt/ M∞ vs. ln t for SRF1 in soil, Figure S16: Graph of Korsmeyer-Peppas of ln Mt/ M∞ vs. ln t for SRF2 in soil, Figure S17: FTIR spectra of blended paper.

## References

[1] Azeem B., KuShaari K., Man Z.B., Basit A., Thanh T.H. (2014). Review on materials & methods to produce controlled release coated urea fertilizer. J. Control. Release.

[2] Jie C., Jing-Zhang C., Man-Zhi T., Zi-tong G. (2002). Soil degradation: A global problem endangering sustainable development. J. Geogr. Sci..

[3] Brown M.E., Hintermann B., Higgins N. (2009). Markets, Climate Change, and Food Security in West Africa. Environ. Sci. Technol..

[4] Mateo-Sagasta J., Zadeh S.M., Turral H. (2018). More People, More Food, Worse Water? A Global Review of Water Pollution from Agriculture.

[5] Azeem B., KuShaari K., Man Z. (2016). Effect of coating thickness on release characteristics of controlled release urea produced in fluidized bed using waterborne starch biopolymer as coating material. Procedia Eng..

[6] Trenkel M.E. (2010). Slow-and Controlled-Release and Stabilized Fertilizers: An Option for Enhancing Nutrient Use Efficiency in Agriculture.

[7] Chen J., Lü S., Zhang Z., Zhao X., Li X., Ning P., Liu M. (2018). Environmentally friendly fertilizers: A review of materials used and their effects on the environment. Sci. Total Environ..

[8] Li W., Guo S., Liu H., Zhai L., Wang H., Lei Q. (2018). Comprehensive environmental impacts of fertilizer application vary among different crops: Implications for the adjustment of agricultural structure aimed to reduce fertilizer use. Agric. Water Manag..

[9] Roshanravan B., Soltani S.M., Rashid S.A., Mahdavi F., Yusop M.K. (2015). Enhancement of nitrogen release properties of urea–kaolinite fertilizer with chitosan binder. Chem. Speciat. Bioavailab..

[10] Ghormade V., Deshpande M.V., Paknikar K.M. (2011). Perspectives for nano-biotechnology enabled protection and nutrition of plants. Biotechnol. Adv..

[11] Tian C., Zhou X., Ding Z., Liu Q., Xie G., Peng J., Eissa M.A. (2021). Controlled-release N fertilizer to mitigate ammonia volatilization from double-cropping rice. Nutr. Cycl. Agroecosyst..

[12] Shoji S. (2005). Innovative use of controlled availability fertilizers with high performance for intensive agriculture and environmental conservation. Sci. China Life Sci..

[13] Chen J., Wei X., Khan A., Fahad S. (2018). Controlled-release fertilizers as a means to reduce nitrogen leaching and runoff in container-grown plant production. Nitrogen in Agriculture-Updates.

[14] Ayub G.S.E., Rocha S.C.S., Perrucci A.L.I. (2001). Analysis of the surface quality of sulphur-coated urea particles in a two-dimensional spouted bed. Braz. J. Chem. Eng..

[15] González M.E., Cea M., Medina J., González A., Diez M.C., Cartes P., Navia R. (2015). Evaluation of biodegradable polymers as encapsulating agents for the development of a urea controlled-release fertilizer using biochar as support material. Sci. Total Environ..

[16] Mulder W.J., Gosselink R.J.A., Vingerhoeds M.H., Harmsen P.F.H., Eastham D. (2011). Lignin based controlled release coatings. Ind. Crop. Prod..

[17] Lum Y.H., Shaaban A., Mohamad N., Dimin F., Yatim N.M. (2016). Boric acid modified starch polyvinyl alcohol matrix for slow-release fertilizer. e-Polymers.

[18] Özen İ., Okyay G., Ulaş A. (2018). Coating of nonwovens with potassium nitrate containing carboxymethyl cellulose for efficient water and fertilizer management. Cellulose.

[19] Zhai L., Park J., Lee J.Y., Kim D., Kim J. (2018). Synthesis, characterization, and antibacterial property of eco-friendly Ag/cellulose nanocomposite film. Int. J. Polym. Mater..

[20] Abdelhamid H.N., Hussein K.H. (2021). Graphene oxide as a carrier for drug delivery of methotrexate. Biointerface Res. Appl. Chem..

[21] Emam H.E., Mohamed A.L. (2021). Controllable Release of Povidone-Iodine from Networked Pectin@ Carboxymethyl Pullulan Hydrogel. Polymers.

[22] Shaheen T.I., Montaser A.S., Li S. (2019). Effect of cellulose nanocrystals on scaffolds comprising chitosan, alginate and hydroxyapatite for bone tissue engineering. Int. J. Biol. Macromol..

[23] Younes H.A., Taha M., Mahmoud R., Mahmoud H.M., Abdelhameed R.M. (2022). High adsorption of sodium diclofenac on post-synthetic modified zirconium-based metal-organic frameworks: Experimental and theoretical studies. J. Coll. Interface Sci..

[24] Emam H.E., El-Shahat M., Abdelhameed R.M. (2021). Observable removal of pharmaceutical residues by highly porous photoactive cellulose acetate@ MIL-MOF film. J. Hazard. Mater..

[25] Radwan E.K., Kafafy H., El-Wakeel S.T., Shaheen T.I., Gad-Allah T.A., El-Kalliny A.S., El-Naggar M.E. (2018). Remediation of Cd (II) and reactive red 195 dye in wastewater by nanosized gels of grafted carboxymethyl cellulose. Cellulose.

[26] Emam H.E., Shaheen T.I. (2019). Investigation into the role of surface modification of cellulose nanocrystals with succinic anhydride in dye removal. J. Polym. Environ..

[27] Abdelhameed R.M., Alzahrani E., Shaltout A.A., Emam H.E. (2021). Temperature-controlled-release of essential oil via reusable mesoporous composite of microcrystalline cellulose and zeolitic imidazole frameworks. J. Ind. Eng. Chem..

[28] Ghosal K., Chakrabarty S., Nanda A. (2021). Hydroxypropyl methylcellulose in drug delivery. Der Pharm. Sin..

[29] Bianchi S.E., Angeli V.W., Souza K.C.B.D., Miron D.D.S., Carvalho G.D.A., Santos V.D., Brandalise R.N. (2011). Evaluation of the solubility of the HPMC: PVA blends in biological fluids in vitro. Mater. Res..

[30] Sofyane A., Ablouh E., Lahcini M., Elmeziane A., Khouloud M., Kaddami H., Raihane M. (2021). Slow-release fertilizers based on starch acetate/glycerol/polyvinyl alcohol biocomposites for sustained nutrient release. Mater. Today Proc..

[31] Gaaz T.S., Sulong A.B., Akhtar M.N., Kadhum A.A.H., Mohamad A.B., Al-Amiery A.A. (2015). Properties and applications of polyvinyl alcohol, halloysite nanotubes and their nanocomposites. Molecules.

[32] Enayatifard R., Saeedi M., Akbari J., Tabatabaee Y.H. (2009). Effect of hydroxypropyl methylcellulose and ethyl cellulose content on release profile and kinetics of diltiazem HCl from matrices. Trop. J. Pharm. Res..

[33] Melaj M.A., Daraio M.E. (2014). HPMC layered tablets modified with chitosan and xanthan as matrices for controlled-release fertilizers. J. Appl. Polym. Sci..

[34] Caccavo D., Cascone S., Amoroso M., Apicella P., Lamberti G., Barba A.A. (2015). Hydrogel-based granular Phytostrengtheners for prolonged release: Production and characterization. Chem. Eng. Trans..

[35] Lum Y.H., Shaaban A., Mitan N.M.M., Dimin M.F., Mohamad N., Hamid N., Se S.M. (2013). Characterization of urea encapsulated by biodegradable starch-PVA-glycerol. J Polym Environ..

[36] Bloom J.M. (2017). Papermaking: The historical diffusion of an ancient technique. Mobilities of Knowledge.

[37] Sahin H.T., Arslan M.B. (2008). A study on physical and chemical properties of cellulose paper immersed in various solvent mixtures. Int. J. Mol. Sci..

[38] Gungula D.T., Andrew F.P., Joseph J., Kareem S.A., Barminas J.T., Adebayo E.F., Ator R. (2021). Formulation and characterization of water retention and slow-release urea fertilizer based on Borassus aethiopum starch and Maesopsis eminii hydrogels. Resul. Mater..

[39] Song P., Wu Y., Zhang X., Yan Z., Wang M., Xu F. (2018). Preparation of covalently crosslinked sodium alginate/hydroxypropyl methylcellulose pH-sensitive microspheres for controlled drug release. BioResources.

[40] Oh C.M., Heng P.W.S., Chan L.W. (2015). Influence of hydroxypropyl methylcellulose on metronidazole crystallinity in spray-congealed polyethylene glycol microparticles and its impact with various additives on metronidazole release. AAPS PharmSciTech.

[41] Somashekarappa H., Prakash Y., Hemalatha K., Demappa T., Somashekar R. (2013). Preparation and characterization of HPMC/PVP blend films plasticized with sorbitol. Indian J. Mater. Sci..

[42] Hermida L., Agustian J. (2019). Slow-release urea fertilizer synthesized through recrystallization of urea incorporating natural bentonite using various binders. Environ. Technol. Innov..

[43] Elhassani C.E., Essamlali Y., Aqlil M., Nzenguet A.M., Ganetri I., Zahouily M. (2019). Urea-impregnated HAP encapsulated by lignocellulosic biomass-extruded composites: A novel slow-release fertilizer. Environ. Technol. Innov..

[44] El Assimi T., Lakbita O., El Meziane A., Khouloud M., Dahchour A., Beniazza R., Boulif R., Raihane M., Lahcini M. (2020). Sustainable coating material based on chitosan-clay composite and paraffin wax for slow-release DAP fertilizer. Int. J. Biol. Macromol..

[45] Wei H., Wang H., Chu H., Li J. (2019). Preparation and characterization of slow-release and water-retention fertilizer based on starch and halloysite. Int. J. Biol. Macromol..

[46] Olad A., Zebhi H., Salari D., Mirmohseni A., Tabar A.R. (2018). Slow-release NPK fertilizer encapsulated by carboxymethyl cellulose-based nanocomposite with the function of water retention in soil. Mater. Sci. Eng. C.

[47] Ni B., Liu M., Lü S. (2009). Multifunctional slow-release urea fertilizer from ethylcellulose and superabsorbent coated formulations. Chem. Eng. J..

[48] Shen Y., Du C., Zhou J., Ma F. (2018). The facile modification of polyacrylate emulsion via hexadecane to enhance controlled-release profiles of coated urea. Sci. Rep..

[49] Liu L., Kost J., Fishman M.L., Hicks K.B. (2008). A review: Controlled release systems for agricultural and food applications. New Delivery Systems for Controlled Drug Release from Naturally Occurring Materials.

[50] Chime Salome A., Onunkwo Godswill C., Onyishi I.I. (2013). Kinetics and mechanisms of drug release from swellable and non swellable matrices: A review. Res. J. Pharm. Biol. Chem. Sci..

[51] Zhang M., Huang Y., Pan W., Tong X., Zeng Q., Su T., Shen J. (2021). Polydopamine-incorporated dextran hydrogel drug carrier with tailorable structure for wound healing. Carbohydr. Polym..

[52] Emam H.E., Shaheen T.I. (2021). Design of a dual pH and temperature responsive hydrogel based on esterified cellulose nanocrystals for potential drug release. Carbohydr. Polym..

