# Supplementary material for: Synthesis and Characterization of Slow-Release Fertilizer Hydrogel Based on Hydroxy Propyl Methyl Cellulose, Polyvinyl Alcohol, Glycerol and Blended Paper
Source: Gels. 2021 Dec 13;7(4):262. doi: 10.3390/gels7040262 (PMC8700842; doi:10.3390/gels7040262)
Supplement: Supplementary file 1 [file gels-07-00262-s001.zip › gels-1512924-supplementary.pdf]

gels MDPI

Article

Synthesis and Characterization of Slow-Release Fertilizer Hydrogel Based on Hydroxy Propyl Methyl Cellulose, Polyvinyl Alcohol, Glycerol and Blended Paper

Semiu A. Kareem [1,*], Idayatu Dere [1], Daniel T. Gungula [2], Fartisincha Peingurta Andrew [3,*], Abdullahi M. Saddiq [4], Elizabeth F. Adebayo [5], Vadlya T. Tame [2], Haruna M. Kefas [1], Japari Joseph [6] and David O. Patrick [1]

Supplementary Data

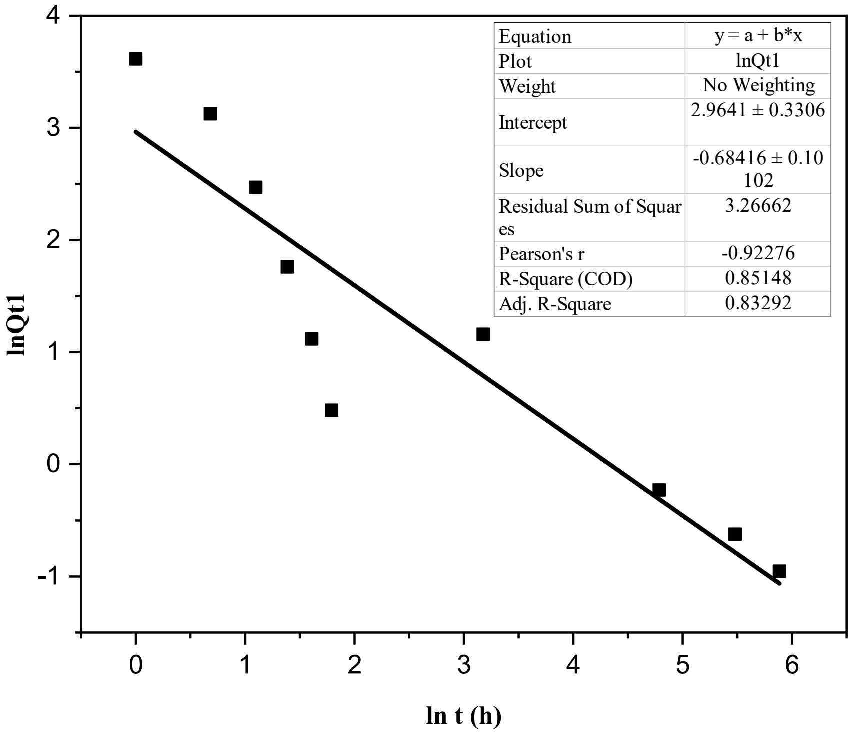

Figure S1. Graph of Zero order kinetic model of ln Q_t vs. ln t for SRF1 in water.

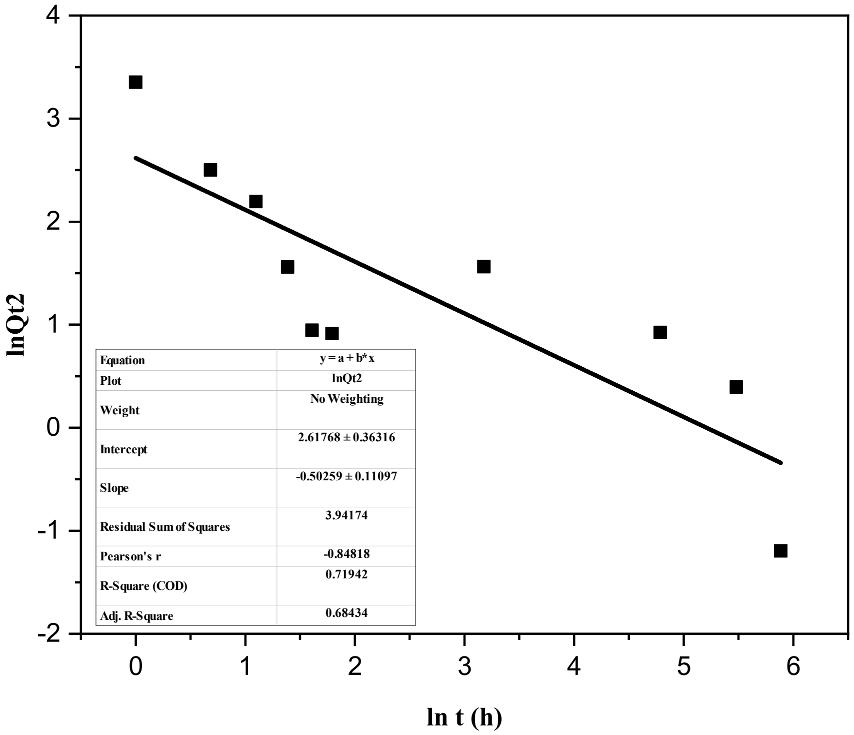

Figure S2. Graph of Zero order kinetic model of ln Q_t vs. ln t SRF2 in water.

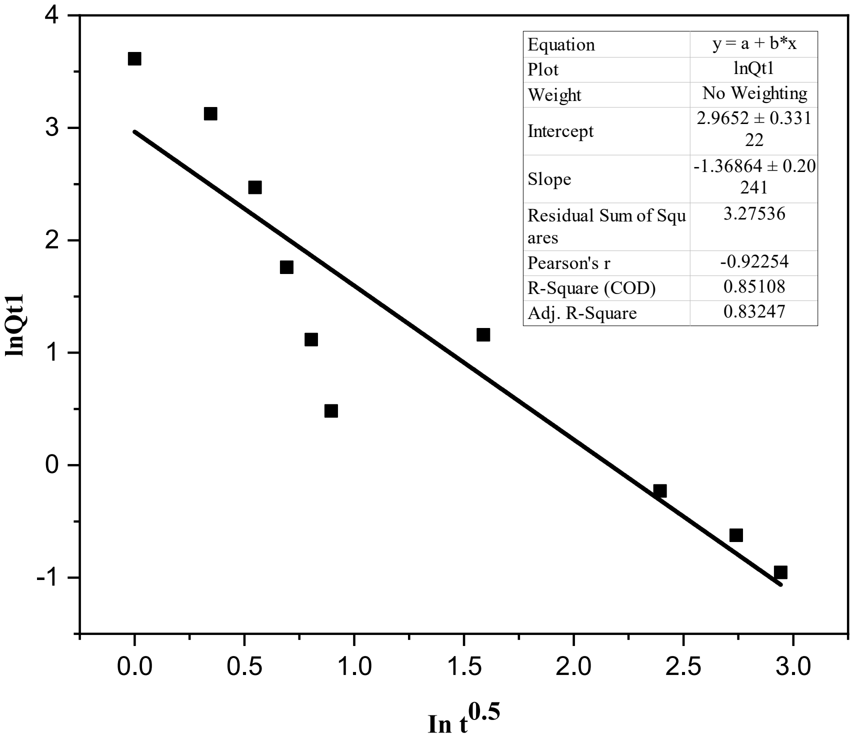

Figure S3. Graph of Higuchi model of ln Qt vs. ln $t^{0.5}$ for SRF1 in water.

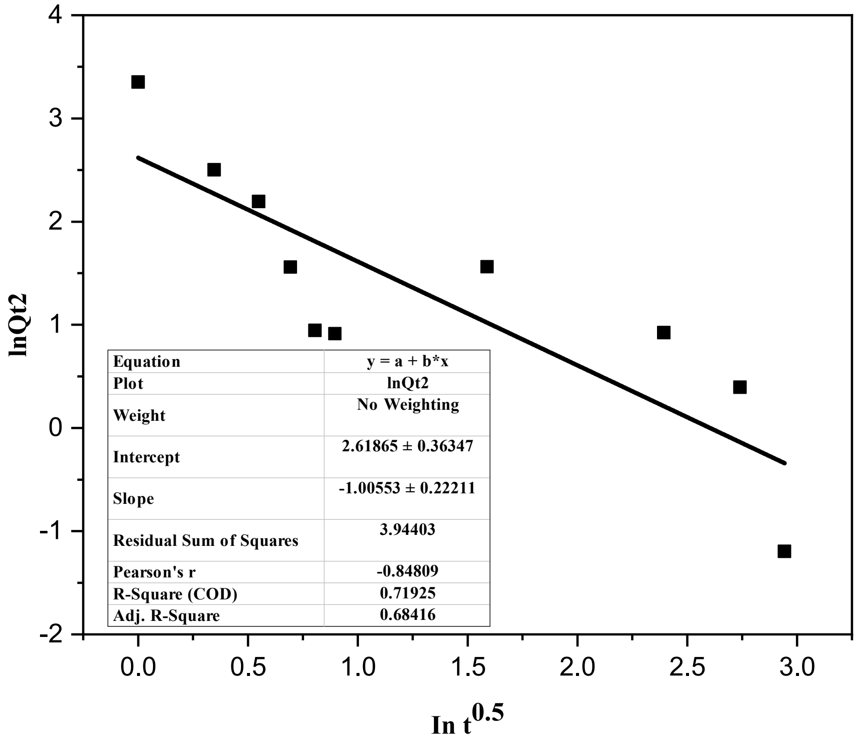

Figure S4. Graph of Higuchi model of ln Qt vs. ln $t^{0.5}$ for SRF2 in water.

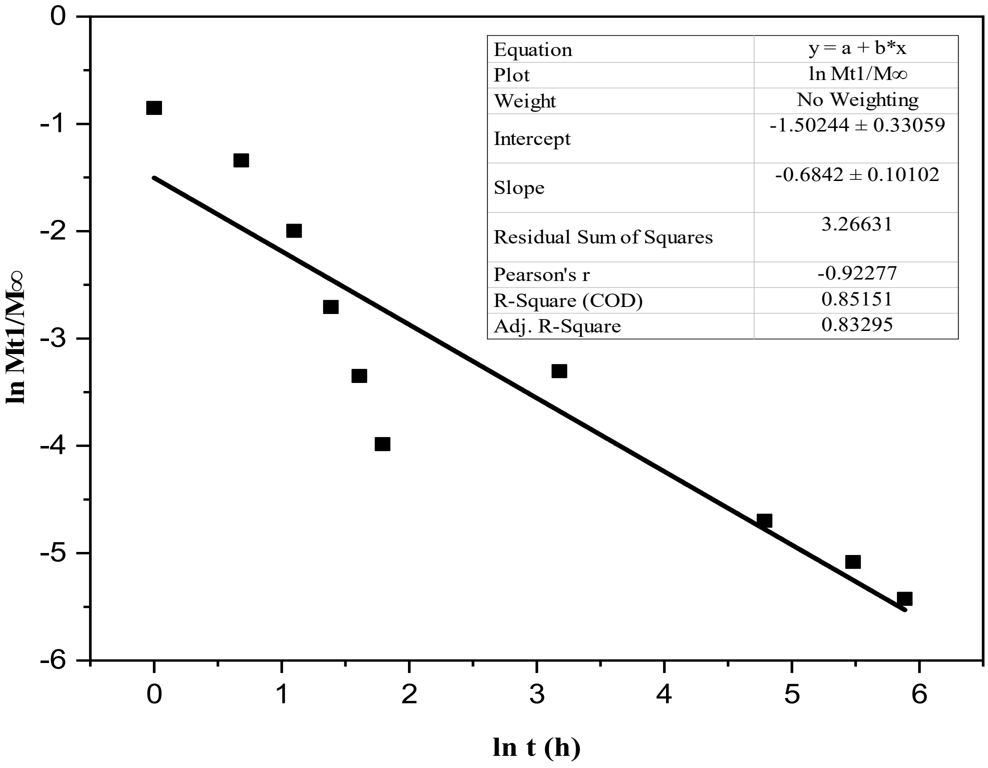

Figure S5. Graph of Korsmeyer-Peppas of ln Mt/M∞ vs. ln t for SRF1 in water.

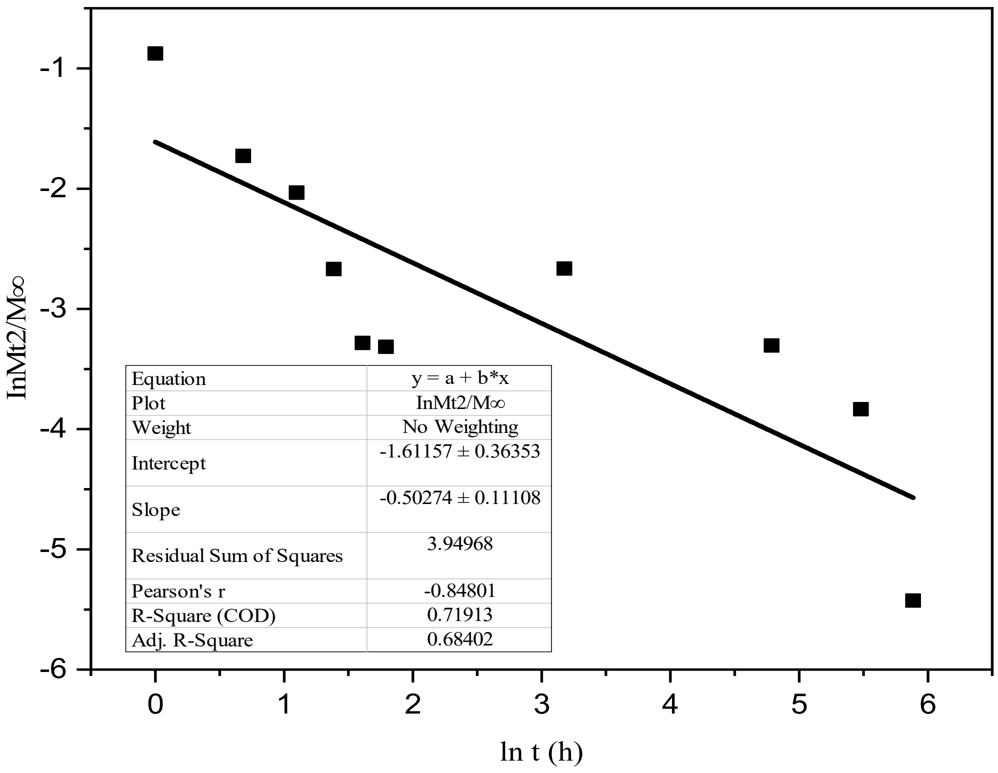

Figure S6. Graph of Korsmeyer-Peppas of ln Mt/M∞ vs. ln t for SRF2 in water.

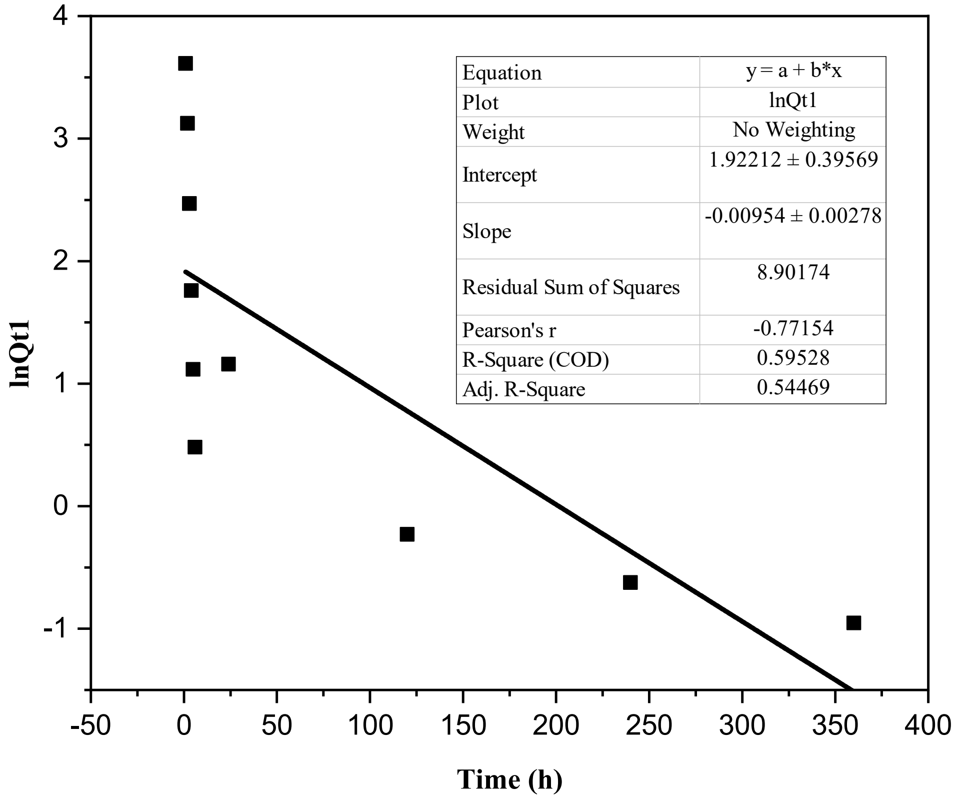

Figure S7. Graph of First order kinetic model of ln Qt vs. Time for SRF 1 in water.

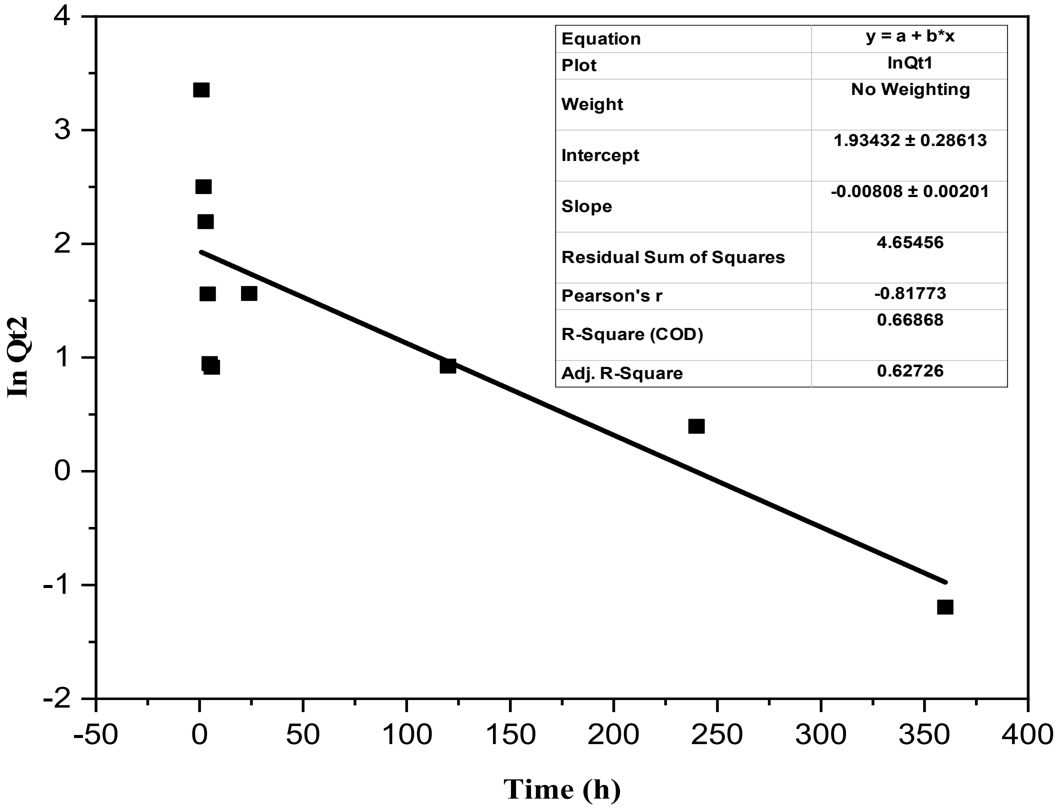

Figure S8. Graph of First order kinetic model of ln Qt vs. Time for SRF 2 in water.

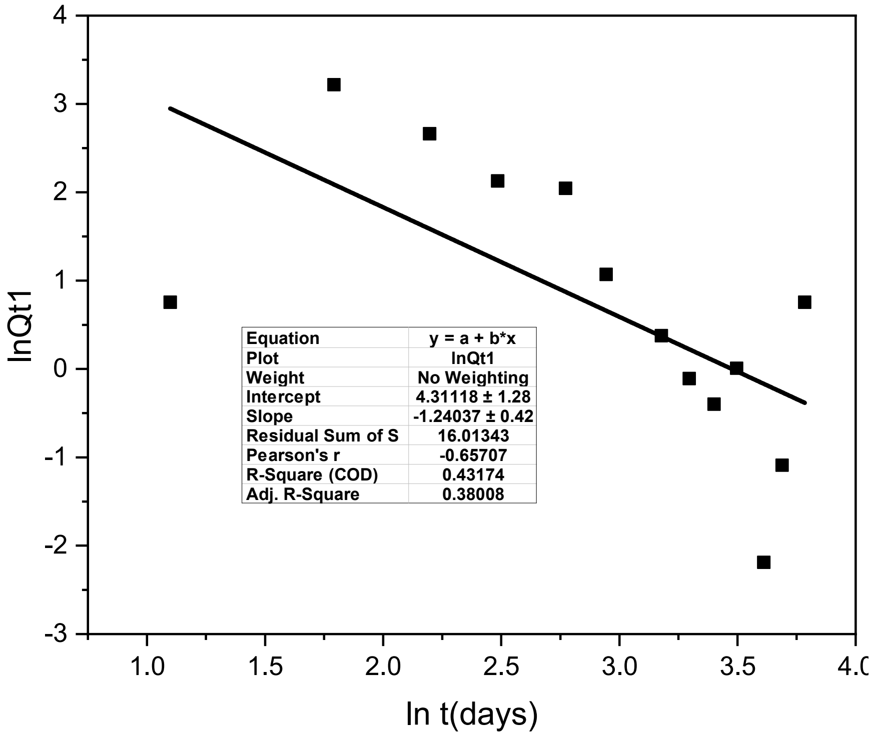

Figure S9. Graph of Zero order kinetic model of ln Qt vs. ln t for SRF1 in soil.

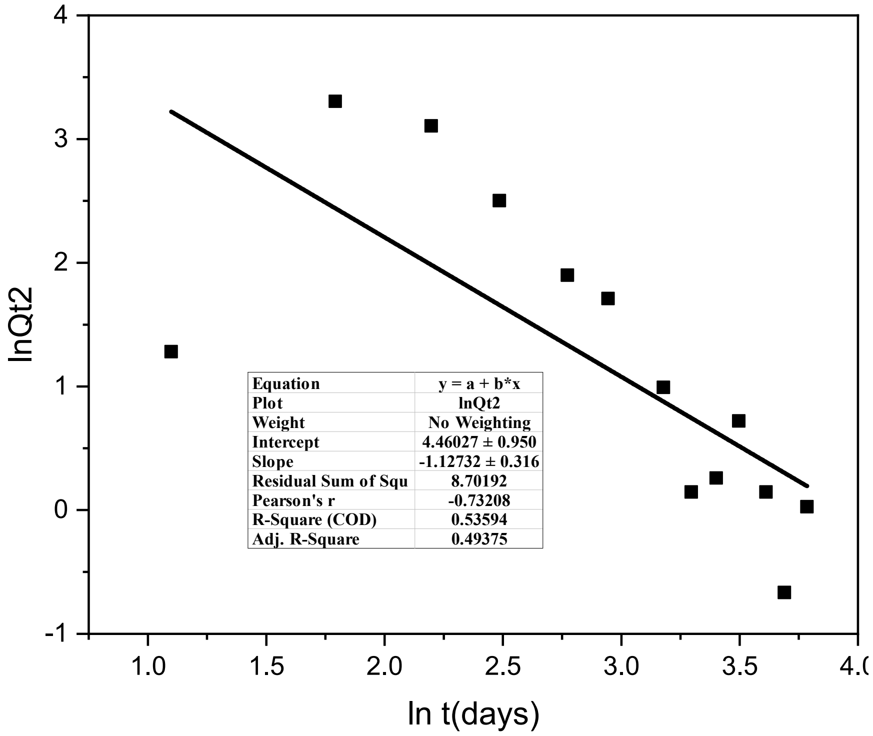

Figure S10. Graph of Zero order kinetic model of ln Qt vs. ln t for SRF2 in soil.

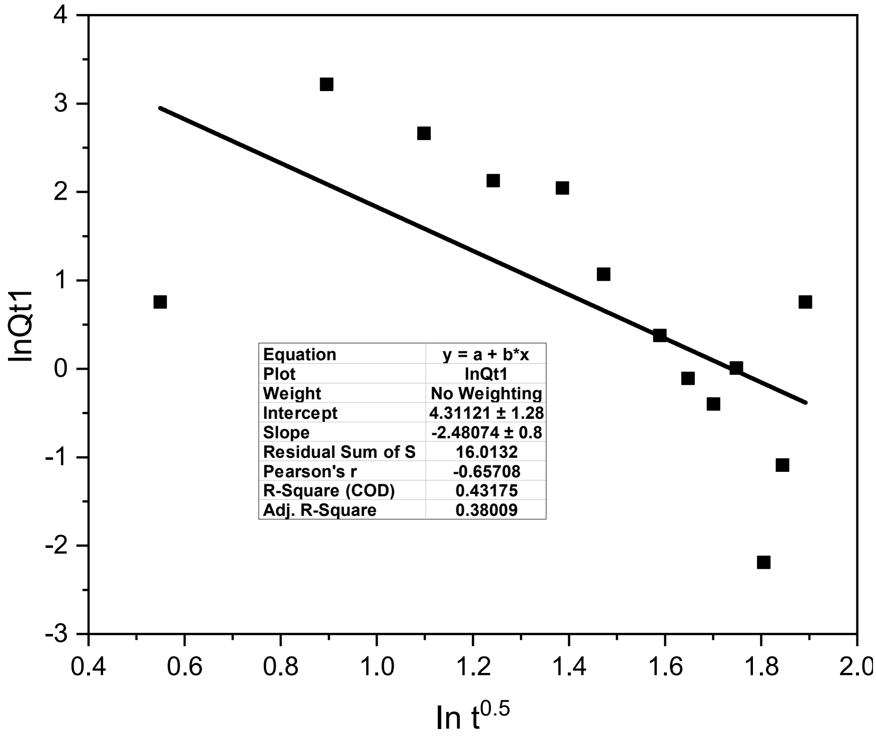

Figure S11. Graph of Higuchi model of ln Qt vs. ln $t^{0.5}$ for SRF1 in soil.

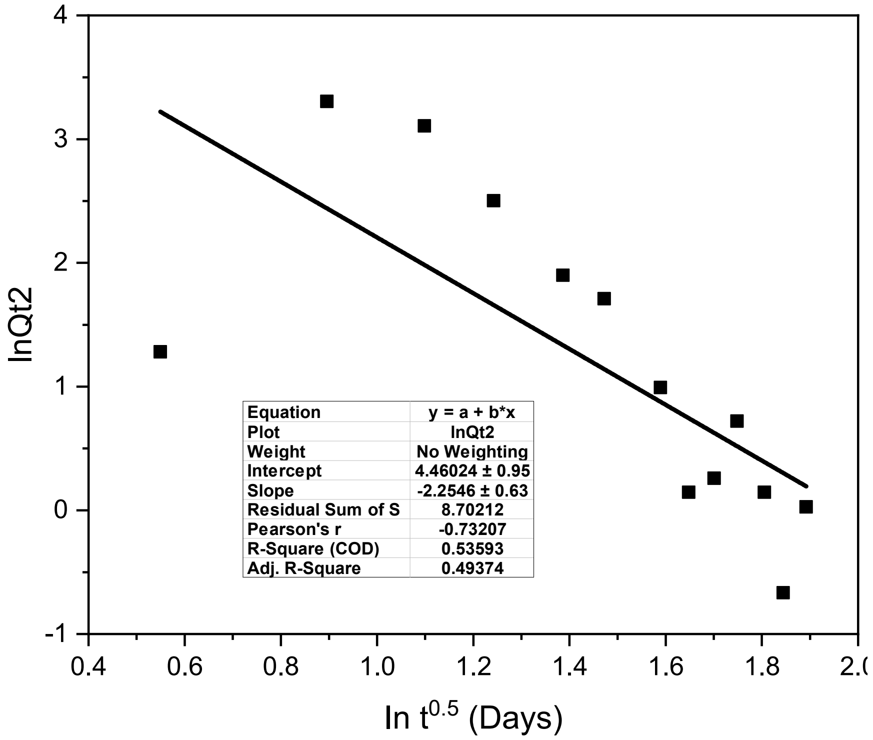

Figure S12. Graph of Higuchi model of ln Qt vs. ln $t^{0.5}$ for SRF2 in soil.

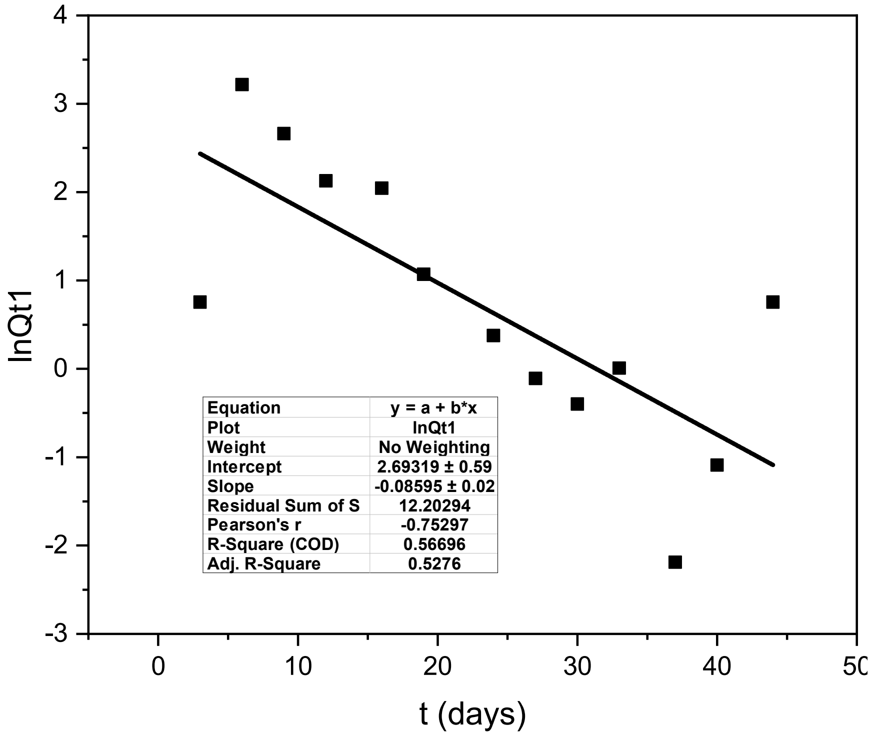

Figure S13. Graph of First order kinetic model of ln Qt vs. Time for SRF 1 in water.

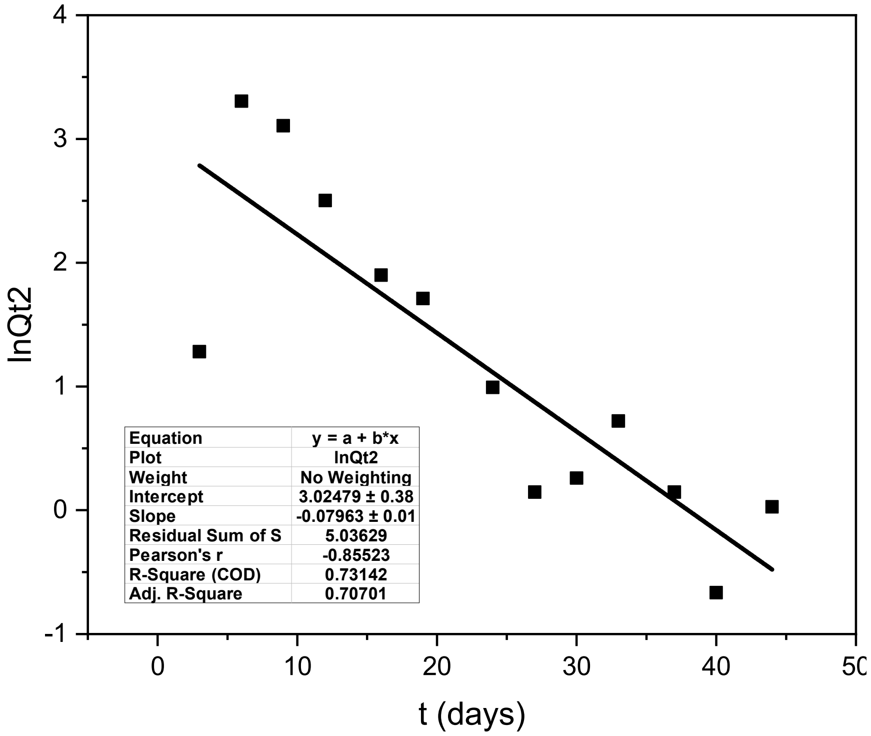

Figure S14. Graph of First order kinetic model of ln Qt vs. Time for SRF 2 in water.

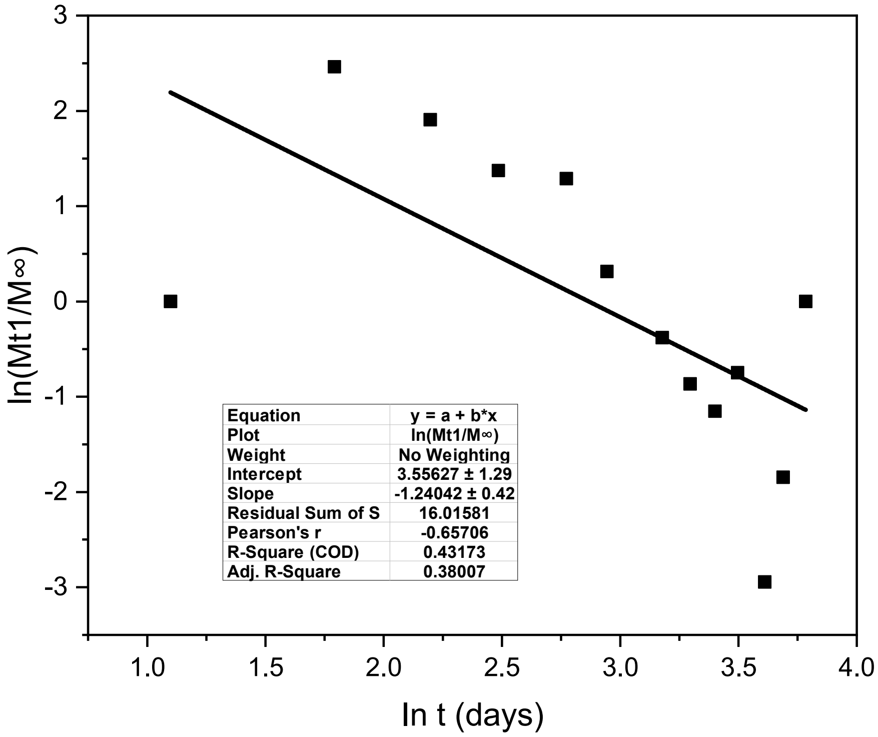

Figure S15. Graph of Korsmeyer-Peppas of ln Mt/ M∞ vs. ln t for SRF1 in soil.

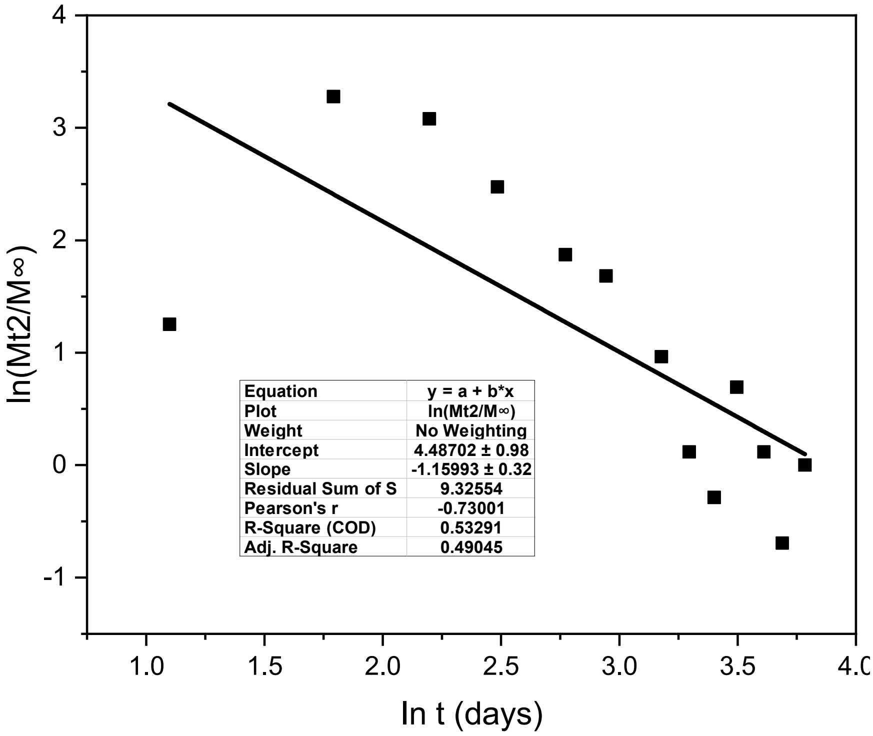

Figure S16. Graph of Korsmeyer-Peppas of ln Mt/M∞ vs ln t for SRF2 in soil.

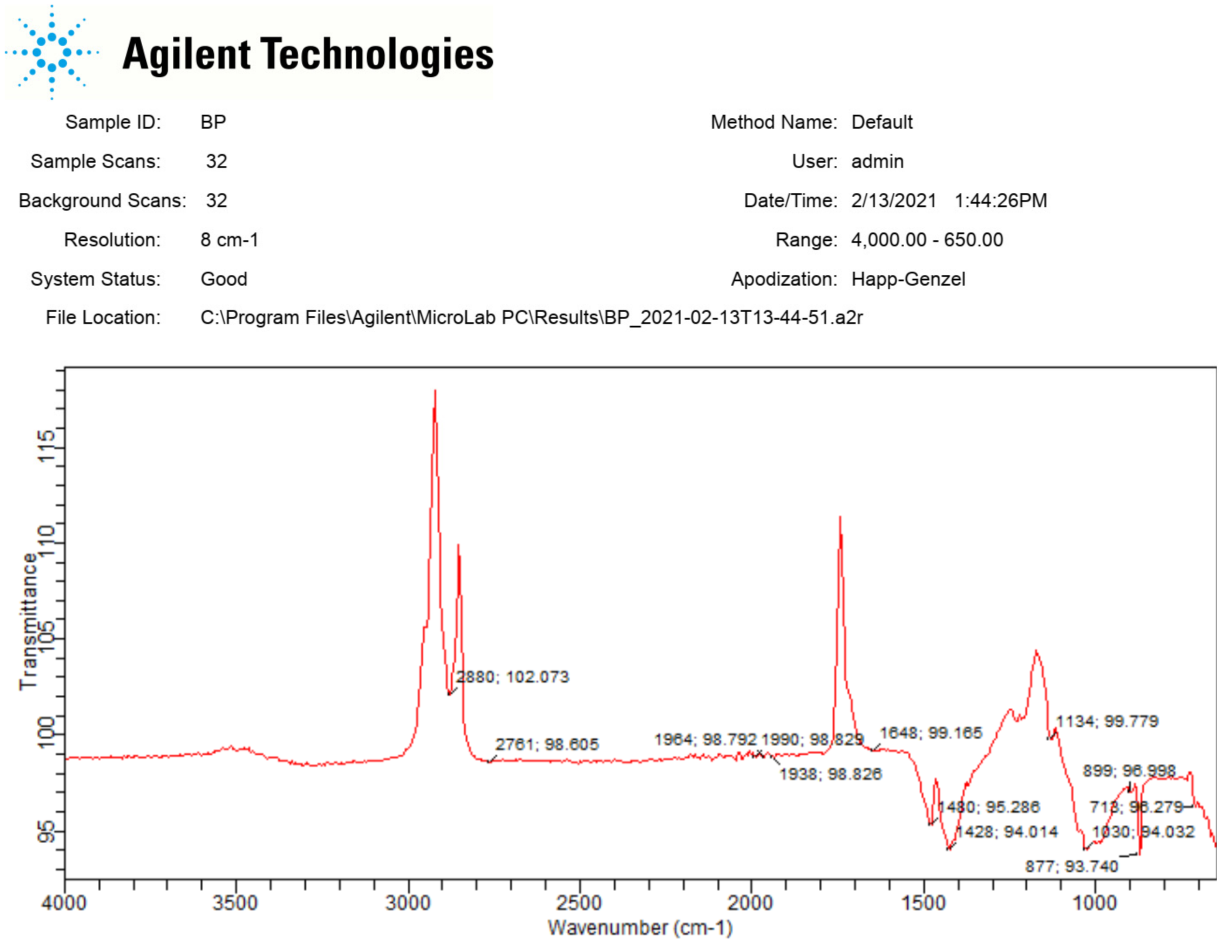

Figure S17. FTIR spectra of blended paper.
